# How Does Workplace Ostracism Lead to Service Sabotage Behavior in Nurses: A Conservation of Resources Perspective

**DOI:** 10.3389/fpsyg.2020.00850

**Published:** 2020-05-26

**Authors:** Ambreen Sarwar, Muhammad Ibrahim Abdullah, Hira Hafeez, Muhammad Ahsan Chughtai

**Affiliations:** ^1^Department of Management Sciences, COMSATS University Islamabad, Lahore Campus, Lahore, Pakistan; ^2^Department of Management Sciences, The University of Lahore, Gujrat Campus, Gujrat, Pakistan; ^3^Department of Management Sciences, Bahria University, Lahore Campus, Lahore, Pakistan

**Keywords:** workplace ostracism, stress, customer service sabotage, perceived organizational support, conservation of resources theory, Pakistan

## Abstract

This article aims to investigate how workplace ostracism acts as a motive behind customer service sabotage. We examine the role of stress as a meditating variable along with the moderation of perceived organizational support (POS) on the said association by using conservation of resources and equity theory. A total of 217 nurses from hospitals of the southern Punjab region in Pakistan participated in the study. Data were collected through survey and structured questionnaires. SPSS and AMOS were used to analyze data with the latest techniques of bootstrapping and process macros. The results showed that stress mediated between the association of workplace ostracism and service sabotage behavior. POS was confirmed as a moderator between this relationship. POS buffered the harmful effects of ostracism and stress on customer service, as POS demonstrates to personnel that they are cherished and respected by the organization. This lessens the strength of perceived stress due to workplace ostracism. Organizational leadership should take advantage of the stress-alleviating effect of POS, which is important in producing adequate levels of work performance.

## Introduction

Workplace ostracism (WPO) is described as an extent to which an individual believes to have sensed the feelings of being ignored, excluded, or barred at workplace by workplace peers (Williams, 2007; Ferris et al., 2008). Several studies have shown instances of employee’s feelings of being ostracized (Fox and Stallworth, 2005; O’Reilly et al., 2014). Ostracism in work settings takes the form of isolation, segregation, leaving the room on entrance of other individual, avoiding eye contact, failing to answer a co-worker’s greetings, and transferring someone to a remote location (Robinson et al., 2013; Xu et al., 2017).

Workplace ostracism is a covert form of mistreatment. When employees come across verbal abuse, rude or indecorous behavior, or discriminatory burden in the workplace, mistreatment arises (Abubakar et al., 2018). In such a situation, mistreatment can exacerbate the mental pressure that employees confront, which might ensue stress. There is an increasing research interest being put to employee mistreatment at workplace (Steinbauer et al., 2018; Wu et al., 2018). A lot of research has demonstrated its detrimental influence on employee’s health, attitude, and behaviors (Jahanzeb and Fatima, 2017; Lyu and Zhu, 2017; Harnois and Bastos, 2018). Recently, researchers (Chang et al., 2019; Howard et al., 2019; Huertas-Valdivia et al., 2019; Sarfraz et al., 2019) have started focusing on the most widely prevailing form of mistreatment—WPO (Fox and Stallworth, 2005; as cited by Steinbauer et al., 2018). Ostracism gives a sense of social rejection and exclusion to employees that can possibly hinder their capability to boost organizational advantages (Jahanzeb and Fatima, 2017). With this understanding of the outcomes of WPO, researchers have shifted their focus to its underlying mechanism (Xia et al., 2019).

In the same vein, the current study utilizes conservation of resources (COR) theory (Hobfoll et al., 2018) and equity theory (Blau, 1964), which offers an ideal opportunity for comprehending the effects of WPO (Xia et al., 2019). WPO reduces worthy resources that are vital to support personnel in their organizations (Leung et al., 2011). In this situation, a person’s defense mechanism would be stirred. In order to defend against additional resource loss, personnel might face continuous stress and suffer more resource harm, resulting in various destructive work-related organizational outcomes.

Furthermore, consistent with the COR theory, personal, situational, and other resources might prove to aid in the reduction of harmful effects of resource loss that may ultimately result in substandard performance. Therefore, we propose perceived organizational support (POS) as an environmental job resource in this study, which might play its role as a significant recovery source and aid in reducing the harmful influence of stress on nurses. A supportive culture of organization is positively associated to commitment of employees to their roles (Lok and Crawford, 2001), along with their work satisfaction and the excellence of customer care that employees provide (Kangas et al., 1999). Earlier, it has been suggested that social support, usually recognized as the emotional and practical resource offered by others, could have an advantageous influence on employee’s health and occupational well-being (Watts et al., 2013). According to Manning et al. (1996), the association of well-being and perceived social support is more striking when people are under more stress.

Among the healthcare professions, nursing is one of the most renowned as a stressful profession (Hunsaker et al., 2015; Martos et al., 2018). Nurses are exposed to uninterrupted instances of emotionally charged-up and challenging circumstances. They have to provide sympathetic, compassionate care usually in unpleasant settings (Bolton, 2001). Nurses are expected to regulate their own feelings and are also assumed to ease up the pain of patients and worry of patient’s families (Diefendorff et al., 2011). The stressful mental state of nurses during job duty can harmfully affect the patient’s care. This can result in substantial medical errors that might become detrimental to patients as well as hospital-related organizational consequences.

For upholding an individual’s well-being and healthiness, restoring from a stressful state is essential (Geurts and Sonnentag, 2006). Without a full restoration, an individual would be susceptible to acute health hazards including hypertension (Hocking Schuler and O’Brien, 1997) and in much severe incidents to cardiovascular death (Kivimäki et al., 2006). Within the premises of COR theory, we believe that WPO might exacerbate stress in nurses, and further, the negative influence of this stress may result in declined performance and/or non-facilitating conducts (Chung, 2018) toward patients including service sabotage behavior—where personnel deliberately trouble the rightful interests of a customer.

According to Harris and Ogbonna (2006), customer service sabotage (CSS) refers to service employee’s misconduct, which is deliberately intended to harmfully influence service. Retaliatory behavior, varying the pace of service, doing mischief, and showing frustration or hostility toward service consumers are illustrations of such service sabotage. In a study conducted by Harris and Ogbonna (2002), among the customer contact personnel, in excess of 85% stated to have been involved in some sort of CSS behavior within a week preceding the study. Also, the same personnel stated that service sabotage happens daily within the workplace.

The role of WPO as an interpersonal stressor (Williams, 1997; Jahanzeb and Fatima, 2017) has been studied earlier, yet its role in aggravating stress (Chung, 2018), leading to service sabotage behavior, and the role of POS as a moderator in the stated association are largely unknown in the sample of nurses (Sarfraz et al., 2019). It is important to comprehend the association among WPO and stress-related outcomes (Wu et al., 2012) for improving the well-being of nurses. Therefore, this study will investigate the association between WPO, stress, and CSS to fill this empirical gap. Moreover, we answer to different calls for identifying various boundary conditions (Lyu and Zhu, 2017; Zhu et al., 2017; Abubakar et al., 2018; Chung, 2018) that may reduce the harmful influence of ostracism, by utilizing theories other than job embeddedness (Lyu and Zhu, 2017) in the service sector (e.g., healthcare, banking, hospitality, and other) of a developing country (Abubakar et al., 2018) since these associations are underexplored (Chung, 2018). We utilize POS as a useful occupational resource that might play the role of a mitigating variable and protect against the damaging consequences of WPO on nurse’s stress in the Pakistani healthcare industry.

## Review of Literature

### WPO and CSS

The sense of exclusion in a person can lead to various negative mental and emotional conditions like annoyance, despair, worry (Anderson and Pulich, 2001; Colligan and Higgins, 2006), isolation and grief Hitlan et al., 2006a, b), emotional exhaustion, anger (Wu et al., 2012), and negative emotions (Gonsalkorale and Williams, 2007). Afterward, these negative mental states induce undesirable job outcomes, for instance, counterproductive work behavior (CWB) (Ferris et al., 2008; Zhao et al., 2013), lesser productivity, and higher level of absenteeism (Colligan and Higgins, 2006).

Additionally, the equity theory (Anderson and Schalk, 1998) provides theoretical support for this relation. The theory and the empirical work by Adams (1963) advocates that inequity in social exchanges derives individuals to make adaptive responses in a number of ways, both behavioral and cognitive. In simple words, the theory suggests that humans are rational beings and that they use the cost–benefit analysis to compare the equality of their and others’ contributions and the outcomes (Chao et al., 2011). Based on the equity theory, if employees do not receive the return that is equal to what they have contributed, they would perceive that the equity is violated. Additionally, when personnel perceive that the equitable exchange is breached, they might decide to re-claim the equity by indulging in misbehaviors, like being absent from work (Chao et al., 2011) or not performing at par. Past research suggest that employees would react much more unfavorably to the organization as a way to raise their equity feeling (Greenberg, 1990). The ostracized personnel are deprived of important social resources that are important for well-being as well as for successful completion of job duties (Imran et al., 2019). These employees perceive that their work efforts are not being equitably rewarded by the organization. They try to restore equity by reducing their contribution toward organizational success that largely depends on its customers. Thus, we propose that:

**Hypothesis 1:** Workplace ostracism is positively related to customer service sabotage.

### The Mediating Role of Stress

Workplace ostracism has a harmful influence on personnel’s well-being since it is an excruciating and disliked experience (Ferris et al., 2008). Researchers have demonstrated that ostracism is related to negative affect (Williams et al., 2002), frustration, anxiety (Anderson and Pulich, 2001; Colligan and Higgins, 2006), emotional exhaustion (Wu et al., 2012), and negative emotional states like sadness, depression, loneliness, jealousy, guilt, and social anxiety (e.g., Gruter and Masters, 1986; Leary et al., 2001). Williams (1997, 2001) proposed that ostracism can be contended as an interpersonal stressor, consequently, ensuing in stress.

The term “stress” was coined by Hans Selye in 1936 (Selye, 1973). According to Lazarus and Folkman (1984), stress is defined as “particular relationship between the person and the environment that is appraised by the person as taxing or exceeding his or her resources and endangering his or her well-being” (p. 11).

Stress is detrimental and triggers a chain of negative emotions and mental states that might lead to withdrawal from work or service production. Negative emotions and moods have been found to be associated with service sabotage (Lee and Ok, 2014; Chi et al., 2015; Abubakar et al., 2018). Stress harmfully affects a person’s efficiency, effectiveness, and his or her quality of performance (Savery and Luks, 2001). People with higher levels of stress encounter cognitive exhaustion, which leads to draining of a person’s energy that he or she requires for task completion. Accordingly, when people encounter stress, they are expected to show deteriorated performance on job duties that require patience, precision, and the ability to concentrate (Motowidlo et al., 1986).

When people are isolated, their cognitive state is harmfully influenced, which curtails self-awareness, highlights the present situation, and reduces thoughts about long-term objectives (Twenge et al., 2003). Consequently, such people would focus more on the present and on mechanisms that might conserve their present energy resources.

Conservation of resources theory most coherently captures the essence of this idea. It states that individuals try to maintain, add, and preserve their valuable resources (Hobfoll, 1989) including belongingness, health, well-being, esteem, family, and a meaningful life (Hobfoll et al., 2018). These resources determine an individual’s personal and communal worth. A sense of threat to these resources may initiate a tend-and-befriend reaction (Williams, 2007) and results in stress (Hobfoll et al., 2018). COR theory posits that “stress occurs (*a*) when central or key resources are threatened with loss, (*b*) when central or key resources are lost, or (*c*) when there is a failure to gain central or key resources following significant effort” (Hobfoll et al., 2018, p.104). In order to conserve the resources, personnel require sharing their thoughts and having emotional associations with their counterparts at the workplace (Heaphy and Dutton, 2008). By adversely impacting personnel’s emotional and mental state (Liu et al., 2013), WPO (as a stressor) depletes the necessary resources required for accomplishing job duties (Wu et al., 2012). People utilize various behaviors deemed to lessen the recurrent exhaustion and preserve their worthy resources required for tackling anxious circumstances (Leung et al., 2011).

In particular, when nurses face demanding situations with patients while in stressful mental state, they might sense emotional and physical exhaustion, reduced energy levels, and excessive fatigue, even feeling too exhausted of emotional resources to deal with continuing demands (Lee and Ok, 2014) of giving excellent patient care. To manage this sense of resource exhaustion and to conserve resources, nurses might end up indulging in service sabotage. Furthermore, when nurses are drained off their capacity for empathy, they get detached from, and insensitive to, the affairs of their patients (Watts et al., 2013).

In light of these arguments, this study predicts a mediating role of stress, such that WPO reduces the quality of patient service experience from nurses due to the increase in nurse’s stress levels. In accordance with COR theory, nurses who perceive greater resource loss due to workplace isolation and exclusion will sense a feeling of stress. In order to maintain the resource conservation process, they will inhibit spending further resources in friendly patient care and therefore exhibit service sabotage behavior (Hobfoll, 2001). Hence:

**Hypothesis 2:** Stress mediates the positive association between WPO and service sabotage.

### The Moderating Role of POS

The POS can be described as an extent to which an individual feels to be treasured and cherished for by the organization in exchange to his/her contributions (Eisenberger et al., 1986). It denotes that an individual’s well-being is being taken care of by the organization in return of one’s work efforts (Rhoades and Eisenberger, 2002). POS is considered as the company’s input in positive reciprocity process with personnel, as they are expected to perform well for returning the courtesies of attained rewards and favorable treatment. POS includes the perceptions of organizational justice, better work environment, rewards, and good supervisory relations that specify to employees that they are appreciated and valued by their organization and offers them a reason to rely on the organization (Rhoades and Eisenberger, 2002).

The current literature has shown that social support has a cushioning influence in the relation between stressors and stress reactions, like anxiety, hopelessness, aggravation, well-being, and productivity (Cohen and Wills, 1985; Kaufmann and Beehr, 1986; Kirmeyer and Dougherty, 1988; Scott et al., 2014; Viswesvaran et al., 1999). WPO signifies a stressor because isolation or social exclusion arouses the fragments of the brain connected to corporal pain (Eisenberger and Lieberman, 2005) and consequently might lead to depression, feelings of misery and solitude (Williams, 2007; Williams and Zadro, 2005).

Employees who sense greater POS would have lesser feelings of being stressed due to ostracism; nevertheless, it may just be present (Venkatachalam, 1995; Robblee, 1998). When POS is lesser, the stress is expected to be sensed in more strength. Likewise, Richardson et al. (2008) found POS to be significantly associated with all the stressor and strains studied in their research.

Furthermore, since support has been regarded as an important social environmental aid for personnel at job (Bacharach and Bamberger, 2007; Bacharach et al., 2008), POS represents a key job resource. It refers to the overall accessibility of work segment colleagues to important items, energy, and social resources offered by their peers at work. It is conceivable that the higher the POS, the rate and extent of resource loss resulting from workplace mistreatment may be reduced and countered by the resource gain from support and therefore the resulting stress would not translate into service sabotage behavior from personnel. A cross-sectional study conducted by Aiken et al. (2002) comprising of approximately 10,000 nurses in critical hospitals across four western countries showed that the observations of poor organizational support were associated directly to low job satisfaction and higher instances of burnout. Thus, the earlier studies have demonstrated that improving perceived support can decrease the harmful effects of burnout and other features of occupational stress (Watts et al., 2013).

Consistent with the COR, POS might prove to be an important resource that facilitates the replenishment of personnel’s depleted resources due to stress and may help cope by decreasing the harmful influence of stress on patients. More specifically, POS can be better viewed as an important job resource that might be activated to counteract with the negative influence (like tension, deviant actions) of initial resource damage due to stressful work settings (Hobfoll, 2002; Hobfoll et al., 2003; Halbesleben, 2006). When personnel senses the danger of energy resource loss from work stressors, POS can work as an important resource to counter the stressful states and can mitigate the effects of such stress on customer services. This discussion results in the subsequent hypothesis:

**Hypothesis 3:** POS moderates the indirect association between WPO and service sabotage behavior through stress such that the association will become weaker in the presence of high POS and vice versa.

**FIGURE 1:**
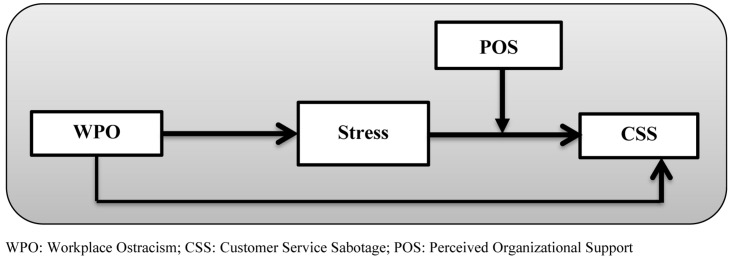
The proposed model of moderated mediation. WPO: workplace ostracism; CSS: customer service sabotage; POS: perceived organizational support.

Figure 1 presents the research model of the study, and demonstrates the proposed hypotheses comprehensively.

## Methodology

### Study Design, Sample Size, and Procedure

Abubakar et al. (2018) have mentioned that in developing nations, personnel employed in the services sector usually bear mistreatment because of the lack of strong legislative structure. Consequently, we selected our population from the healthcare industry, consisting of nurses working in Pakistan’s public sector hospitals in the southern Punjab region. It is vital to verify that organizational backgrounds chosen for investigations mirror the attitudes and actions relevant to the fundamental area of interest and are involved for measurement in the research model (Zhou and George, 2001). Because of the routine, daily contact of nurses with their colleagues and patients, and keeping in mind the highly stressful environment of hospitals owing to the amount of work, task interdependence, ill treatment, and inharmonious relationships, nurses were selected to be studied for this research.

Sample was randomly drawn from hospitals through convenience sampling technique. Survey technique with self-administered paper-and-pencil questionnaires in the English language was used, because it is the official language of communication in most Pakistani organizations and education institutes (De Clercq et al., 2018).

Initially 242 nurses participated in the study. However, after data cleansing, the sample size was reduced to 217 (151 males and 66 females). These nurses responded to demographic, and WPO questions at T1. After a temporal gap of 2 weeks, they filled stress, POS, and service sabotage-related questions at T2. This ensured that the study was least affected by common method bias (Podsakoff et al., 2012). All scales included response ranges from 1 (“strongly disagree”) to 5 (“strongly agree”) unless otherwise mentioned.

Before the commencement of data collection, the participants provided their written informed consent and were updated about the ethical considerations adopted in the study. In order to ensure openness/honesty in responses, anonymity was guaranteed, and respondents were assured that the raw data would not be shared on any platform. This was done for coping with the common method bias (Podsakoff et al., 2003, 2012).

### Instruments

#### Workplace Ostracism

Workplace ostracism was assessed by utilizing a five-point Likert scale created by Ferris et al. (2008), which contains 10 items (e.g., “Others ignored me at work”). The participants answered each question on options on the scale of 5 (i.e., 1 representing “strongly disagree” and 5 representing “strongly agree”). Just like earlier studies (e.g., Chung, 2018), the instrument demonstrated a good reliability (Cronbach’s α = 0.70).

#### Stress

Perceived stress was evaluated by using a seven-item measure developed by Galinsky et al. (1998). The scale consisted of five-point Likert options ranging from never to very often. The statements asked about how frequently over the last 3 months the respondents felt (e.g., “nervous or stressed,” “frustrated by work”). The scale showed a good reliability for the present research (Cronbach’s α = 0.74).

#### Service Sabotage

Customer service sabotage was assessed by utilizing the scale developed by Chi et al. (2015) who adopted it from Chi et al. (2013) and Harris and Ogbonna (2006). The scale consists of six items, responded on a five-point Likert scale. The options ranged from Never (1) to Always (5). The items included statements like how often you engage in these types of behaviors during your patient care encounters, e.g., “Intentionally slows down service when you want to.” For this study, service sabotage scale showed alpha reliability of 0.73.

#### Perceived Organizational Support

Perceived organizational support was measured with the eight-item scale from Eisenberger et al. (1986). It has an equal number of positively worded and reverse-coded questions. Example of questions are “This organization cares about my well-being,” and “This organization shows very little concern for me.” Earlier researchers have demonstrated good reliability of the scale as shown by 0.89 (Dawley et al., 2010). For the present study, the scale reliability was 0.83.

#### Control Variables

Age and work experience were controlled for since earlier researchers have revealed them as influencers of ostracism and burnout-related outcomes and have controlled them in recent studies (Lee and Ok, 2014; Chung, 2018). This ensures that the associations between variables are not confounded.

## Data Analysis

Before proceeding on to test the main hypotheses of the study, we conducted some preliminary tests to validate the data with respect to variance, correlation, and descriptive statistics with the help of SPSS. Later, in order to check the support for main hypotheses, hierarchical regression analysis along with bootstrapping techniques and Hayes’s Process macros were utilized. Usually for testing moderated mediation, this technique examines if the value of the moderator influences the extent of the mediation effect (Hayes, 2017). For analyzing significance of the effects, bootstrapping method was utilized for getting robust standard errors for parameter estimation (Hayes, 2017). This procedure generated 95% bias-corrected confidence intervals (CIs) of these effects. The resampling value of the data was set to 5000 resamples. The effects are considered to be significant at α value of 0.05 when the CIs do not include zero.

### Descriptive and Correlation Analysis

The descriptive statistics of participants demonstrated that most of the participants belonged to the age group of 26–35 years with a deviation of 1.02. Moreover, most of them (110; 50%) had more than 6 years of work experience. Majority of respondents (165; 76%) had graduation degrees, and comparatively more participants were female (179; 82%) in comparison to males (38; 18%), representing female segregation in the nursing profession in Pakistan. The figures in Table 1 display a moderate correlation between the variables that are congruent to the standards (Cohen et al., 2014).

**TABLE 1:** Correlation analysis.

Constructs	Mean	SD	A	WE	WPO	ST	POS	CSS
Age (A)	2.16	1.01	1					
Work experience (WE)	5.29	0.49	0.79*	1				
Workplace ostracism (WPO)	3.41	0.72	0.22**	0.19*	1			
Stress (ST)	3.22	0.71	–0.13	0.20*	0.35**	1		
Perceived organizational support (POS)	3.31	0.84	0.10	0.05	−0.40*	−0.59**	1	
Customer service sabotage (CSS)	2.45	0.77	–0.09	–0.06	0.48**	0.50**	−0.30*	1

N = 217, age and work experience are control variables, *p < 0.05, **p < 0.01.

### Confirmatory Factor Analysis

In order to check the validity and model fit indices, confirmatory factor analysis (CFA) was carried out with the help of AMOS-Version 21. The test was done to make sure the instrument was suitably utilized considering the context of the study because it is important to generalize the results (Hoyle, 1991). First, factor loading estimates were calculated through AMOS. The current study included four latent variables: WPO having 10 items, stress with 7 items, POS with 8 questions, and service sabotage with 6 items. The items having the loading of less than 0.6 were deleted and then CFA was performed. The factor loadings of the remaining items are shown in Table 2.

**TABLE 2:** Standardized loading estimates (SLE).

Construct	Items	SLE
WPO	My peers left the area when I entered.	0.63
	I involuntarily sat alone in a crowded lunchroom at work.	0.75
	I noticed others would not look at me at work.	0.75
	My peers at work shut me out of the conversation.	0.78
	My peers at work treated me as if I were not there.	0.71
Stress	I felt nervous and stressed.	0.74
	I felt emotionally drained from my work.	0.88
	I felt burned out or stressed by my work.	0.84
	I felt frustrated by my work.	0.89
POS	This organization values my contribution to its well-being.	0.69
	This organization really cares about my well-being.	0.80
	This organization cares about my general satisfaction at work.	0.86
CSS	I behave negatively toward customers.	0.63
	I intentionally hurry customers when I want to.	0.85
	I mistreat customers deliberately.	0.79
	I intentionally slows down service when I want to.	0.78

*WPO: workplace ostracism; CSS: customer service sabotage; POS: perceived organizational support.*

For completing the CFA, guidelines set by McArdle (1996) were followed. It is appropriate to reach at model fit indices step by step based on the standards present in the work of Hooper (Hooper et al., 2008). The fit indices were reached by following Byrne (2013). The CFA results demonstrated that the constructs are reasonably operationalized and measure what is intended to be measured. The model fit indices displayed appropriate values within acceptable ranges, i.e., RMSEA = 0.07 < 0.08; Chi-square/df = 2.06 < 3.00; and GFI = 0.904; CFI = 0.926; IFI = 0.927; TLI = 0.91 > 0.90. Moreover, the values of average variance explained (AVE), which measures the convergent validity, was also calculated. All the figures were above the minimum acceptable value of 0.5.

For checking the reliability of scales, Cronbach’s alpha values were calculated. Various scholars have provided different acceptable values of Cronbach’s alpha, for example, more than 0.70 (Murphy and Balzer, 1989), and more than 0.5 (Nunally, 1978). For this study, all the scales were found to be reliable with alpha values exceeding 0.7, i.e., WPO = 0.83, POS = 0.70, CSS = 0.85, and Stress = 0.80.

### Common Method Bias

Because the data for all the variables were collected from the same respondents, there was a chance of data being affected by common method bias. The statistical method used to ensure the absence of such bias was Harman’s single factor test. In this regard, the total variance was checked by a single factor model through exploratory factor analysis, in which maximum variance explained by the model was just 25.6%, which is less than 50% (Harman, 1976; Podsakoff and Organ, 1986), thus strengthening our belief that the data were free from common method bias.

### Hypotheses Testing

#### Test of Direct Relation

In order to test hypotheses 1, hierarchical regression test was applied. The results showed support for our first hypothesis, which predicted a positive relationship between WPO and CSS. The numbers (β = 0.58, *t* = 10.9, *p* < 0.001) in Table 3, Model 1 indicate that as the instances of WPO increases, the occurrence of service sabotage behavior from employees would also increase.

**TABLE 3:** Mediating effect of stress between WPO and CSS using process macro.

	Model 1 (path c)			Model 2 (path a)			Model 3 (path b and c′)		
	CSS			Stress			CSS		
Predictor	β	*t*	CI	β	*t*	CI	β	*t*	CI
Age	0.03	0.62	−0.07, 0.1	–0.08	–1.2	−0.2, 0.05	0.06	1.3	−0.03, 0.1
WE	0.007	0.16	−0.08, 0.1	0.16**	2.6	0.04, 0.28	–0.05	–1.1	−0.1, 0.03
WPO	0.58*	10.9	0.17, 0.67	0.27*	7.58	0.13, 0.40	0.47*	9.85	0.38, 0.57
Stress							0.36*	7.62	0.26, 0.45
*R*^2^	0.36*			0.10*			0.49*		
*F*	39.99			7.52			52.54		

Each column is a regression model that predicts the criterion at the top of the column. *p < 0.001; **p < 0.01; bootstrapping at 5000; WE: work experience.

#### Test of Mediation Effect

Hypothesis 2 of our study predicted that stress mediates the relationship between WPO and CSS. To test this mediated relationship, Preacher and Hayes’s (2004) technique was executed at 5000 bootstrapping, by utilizing the Process macro (Hayes, 2013). This test generates CIs for indirect effects. Therefore, this technique minimizes the potential statistical power problems that may arise from asymmetric and other non-normal sampling distributions (MacKinnon et al., 2004). The CI for the indirect effect of WPO on CSS through perceived stress did not include 0 (0.05; 0.15) in support of the presence of mediation.

Regression results presented in Table 3 contain three models to explain the statistics extracted for mediation analysis. Model 1 shows that WPO significantly predicted CSS and 36% variation found in CSS was due to WPO (*R*^2^ = 0.36, *F* = 39.99, *p* < 0.001). Next, Model 2 demonstrates a positive relation between WPO and stress with *R*^2^ value of 0.10. In Model 3, after controlling for WPO, stress positively impacted CSS with an *R*^2^ value of 0.49. Finally, biased-corrected percentile bootstrap method using model 4 of PROCESS macro by Hayes (2017) indicated that indirect path of WPO on CSS via stress was satisfied [*b* = 0.10, SE = 0.025, 95% (0.05, 0.15)]. Therefore, Hypothesis 2 was accepted, supporting the mediation effect of stress between WPO and CSS.

#### Test of Moderated Mediation

The last hypothesis of the study was to investigate the moderating role of POS in the indirect relation between WPO and CSS via stress. To examine this moderated mediation relationship proposed in Hypothesis 3, we applied Preacher et al.’s (2007) procedure and Hayes’s (2013) Process macro, i.e., Model 14 of Hayes (2017). This procedure produces CI for the conditional indirect effects (MacKinnon et al., 2004). The results from the analysis showed that the 95% bootstrap CIs for the conditional indirect effect of WPO on CSS at the low (−1 SD) as well as high level (+1 SD) of the moderator POS did not contain zero (0.08; 0.23 and.02;0.14, respectively). This interaction term for moderated mediation as shown in Model 3 of Table 4 was found to be significant (β = −0.17, *t* = –2.3, *p* < 0.001) with a Δ*R*^2^ = 0.015, *F* = 6.36, *p* < 0.05. The *R*^2^ value for the overall model was also found to be significant (i.e., *R*^2^ = 0.50, *p* < 0.001). Moreover, the index of moderated mediation (*i* = −0.07) and its corresponding CI did not include zero (−0.11, −0.02), showing that POS acts as a mitigating agent against the detrimental indirect effect of WPO on customer services, through perceived stress, in support of Hypothesis 3 and this study’s overall framework.

**TABLE 4:** Moderating effect of POS using process macro.

	Model 1			Model 2			Model 3		
	CSS			Stress (St)			CSS		
Predictor	β	*t*	CI	β	*t*	CI	β	*t*	CI
**Age**	0.04	0.82	−0.05, 0.1	–0.05	–0.94	−0.1, 0.06	0.05	1.1	−0.03, 0.1
**WE**	−0.1**	–2.0	−0.1, −0.002	0.15	3.03	0.05, 0.25	–0.06	–1.5	−0.1, 0.01
**WPO**				0.39*	6.18	0.21, 0.49	0.47*	8.9	0.37, 0.58
**Stress**	1.51*	4.27	0.91, 2.3				0.88*	3.61	0.39, 1.2
**POS**	0.39**	1.48	0.81, 0.95				0.21**	4.5	0.09, 0.28
POS*St	−0.29*	–3.11	−0.49, −0.13				−0.18*	–2.3	−0.3, −0.04
***R*^2^**	0.41*			0.17*			0.50*		
***F***	32.29			15.7			38.18		

Each column is a regression model that predicts the criterion at the top of the column. **p < 0.005; *p < 0.001; bootstrapping at 5000; WE: work experience.

**FIGURE 2:**
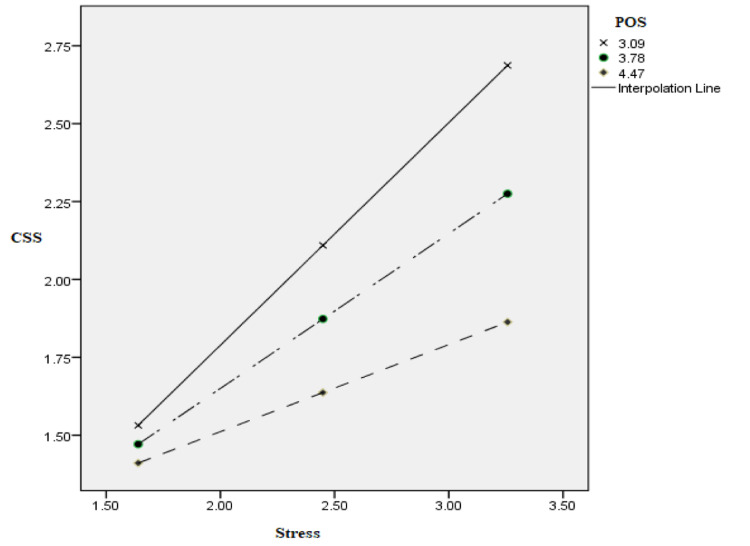
Moderating effect of POS in the relation between stress and CSS.

A simple slope test showed that at different values of POS, the effect of stress on CSS behavior varies significantly; for example, at high level of POS, the effect of stress on CSS becomes weaker (*b* = 0.28, *t* = 3.77, *p* < 0.001, CI = 0.13, 0.42) as compared to low values of POS where the effect of stress on CSS becomes higher (*b* = 0.71, *t* = 7.0, *p* < 0.001, CI = 0.51, 0.91). The significance of the interaction is further proven by plotting the values in a graph with moderating variable at +1 SD and −1 SD. Figure 2 shows the effect of stress on CSS at high, medium, and low levels of POS. The harmful effect of stress in the form of CSS is mitigated in the presence of POS. Therefore, the effect by which stress increases the chances of CSS behavior is buffered by POS.

## Discussion

Mistreatment has emerged as a big problem in contemporary work settings. Its negative effects influence not only organizational outcomes but also employees and their health in the form of deviant behaviors, anxiety, emotional exhaustion, and turnover. This study has extended earlier researches on WPO by showing its link to nurse’s mental health as a result of subsequent stress. Earlier, stress has been studied as an antecedent of negative outcomes (Chung, 2018), yet its relationship with WPO has seldom been studied from a stress viewpoint (Wu et al., 2012; Chung, 2018) in nurses’ sample (Sarfraz et al., 2019). The COR and equity theory provided perfect avenues to study our cause. COR, being a stress theory, explains the human motives for gaining and maintaining vital resources. Because WPO drains resources essential for progressing (Leung et al., 2011), it might trigger a person’s defense mechanism. To guard against additional resource loss, employees encounter constant stress and face increased resource loss, ensuing in a variety of harmful work-related outcomes. Additionally, in line with equity theory, when employees perceive an inequity between the input in and output of their work, they are expected to indulge in counterproductive behavior in an attempt to restore equity.

Our results have demonstrated that workplace (hospital wards) has a social environment that has consequences on the mental and emotional well-being of the nurses. Previous scholars have shown a positive link between WPO and emotional exhaustion (Wu et al., 2012), anxiety (Ferris et al., 2008), and negative effect (Williams et al., 2002). We have not only validated the work carried out by Chung (2018) in Pakistani healthcare context by showing that WPO is positively associated with perceived stress but have also extended it by demonstrating its link to detrimental organizational consequences in the form of CSS and boundary condition of POS. Isolation and silent treatment can create negative self-perceptions, which enables a person to sense resource loss (for example, belongingness). Social support has been contended as a vital resource to tackle stressful situations (Hobfoll, 1989). When employees sense ostracism, which signals isolation from peers and colleagues, a resource loss is perceived. In order to protect themselves from further resource loss, they indulge in counterproductive behavior, which, in our study, surfaced as CSS.

Moreover, people from collectivistic cultures consider harmonious interpersonal connections as important (Yang, 1993). They would be more vulnerable to WPO (Powell et al., 2009). Pakistan is characterized by a comparatively highly power distant, collectivist, and uncertainty avoidant culture, which means that it has a high rule orientation and encourages high disparities in power and affluence (Hofstede, 2001). This cultural sketch represents submissive relations between personnel and employers and indicates a complete respect for power (Hofstede, 1991; Khilji, 1995). This description suggests that personnel would willingly swallow any distress instigated by workplace mistreatment without any retaliatory acts toward their supervisor or colleagues. This continuing endurance, without any aggressive reaction, might transform into a more stressful mental state leading to anger toward customers (whom they do not know), in the form of service sabotage.

Interestingly, little work has been done in the services sector of developing nations (Abubakar et al., 2018), specifically the healthcare industry, regarding workplace mistreatment where legislative protection for such behavior is minimal. Hence the current research provided useful insights into nurses employed in a developing nation with collectivistic, risk-averse, and power distant culture.

An additional important contribution of the current research is the authentication of the moderating role of POS in the indirect relation between WPO and service sabotage behavior via stress. The moderated mediation results further extend the existing and mixed literature on POS (e.g., Kirmeyer and Dougherty, 1988; Iwata and Suzuki, 1997; Beehr et al., 2000; Beehr et al., 2010) and on the boundary conditions for WPO and its consequences (Chung, 2018; Koay, 2018). Moreover, the study also answers the call for recognition of more moderators, which might decrease or aggravate the negative behavioral effect of WPO (e.g., Balliet and Ferris, 2013; Ferris et al., 2015).

Consistent with the main propositions of COR theory, when individuals sense ostracism, they will feel a deficiency of belongingness from peers and managers and consequently a resource loss is sensed since social support is an important resource in unpleasant environments (Hobfoll, 1989). In such a case, job resource of POS would be principally valuable in reducing the stress that personnel feel because of the ill treatment in the workplace. The greater is the perception of organizational support, the less would be the likelihood of them feeling overburdened because of emotional/mental drain since they trust the organization as their major beneficiary for improving their well-being (Rhoades and Eisenberger, 2002). Therefore, lesser stress would be sensed because better POS is beneficial and permits employees to concentrate on the effective completion of their job responsibilities instead of being preoccupied in workplace anti-social context.

### Theoretical Contributions

Our proposed moderated mediation model was endorsed by the COR and equity theory in the context of the Pakistani healthcare industry, with WPO as an independent variable, stress as mediator, POS as moderator, and service sabotage as the outcome variable. The tenets of the COR theory put forward that stress arises when (1) valuable resources are feared to be lost; (2) valuable resources are lost; or (3) valuable resources are not attained despite efforts (Hobfoll et al., 2018, p. 104). Our study has provided proof that WPO is sensed as a perceived threat to valuable resources (e.g., belongingness and social support) and thus results in stress. If such stressful work situations are not taken care of, they would result in decreased job performance and counterproductive behaviors.

Additionally, the equity theory endorsed our main argument that ostracized nurses are more likely to behave negatively toward patients. Employees exchange and reciprocate resources only if they are treated well (Imran et al., 2019). Those who come across positive treatment in the workplace are inclined to reciprocate it with positive organizational behaviors, and vice versa (Li and Tian, 2016). However, ostracized nurses are deprived of important social resources, which are important for their well-being as well as for successful completion of their job duties, and therefore, in return, they reciprocate by reducing their contribution toward organizational success that largely depends on their patient’s satisfaction.

The study also used POS as an important work resource in accordance with COR for reducing the negative affect of resource loss due to WPO. POS signifies support from organization in the shape of justice, rewards, job settings, and supervisory relations (Rhoades and Eisenberger, 2002), all of which are positive resources for the personnel, which show them that they are cared for and appreciated by their organization. When personnel sense this support, they are likely to report having access to the resources essential for work and are motivated to trust their organization for aiding in the improvement of their well-being (Rhoades and Eisenberger, 2002). Such support makes individuals less prone to sense stress in the face of mistreatment in the premises of a supportive organization. They focus and dwell more on organizational resources and lesser on others’ behavior that results in stress.

### Practical Implications

Since this research demonstrates that WPO and stress are related, organizations should attempt to reduce occurrence of WPO. However, recognizing the presence of WPO could be a demanding task as it is a covert and sometimes subjective form of mistreatment. Personnel would also be reluctant to report WPO for the fear of being marked as susceptible or chronic complainants (Estes and Wang, 2008).

Organizations must continually search for innovative ways of identifying the prevalence of WPO and study its fundamentals, including work overload, undesirable role models or communication manners (Pearson and Porath, 2005). Custom-made training can be arranged that highlight the importance of cooperation, both inside and across departmental boundaries. Firms may also encourage personnel for frequent social interaction and offer opportunities for mingling with each other. If employees get opportunities to have pleasant and agreeable time with each other, there would be less instances of WPO. For supervisors, training programs that aid their supportive skills (like open-door strategies, emotional suppression, mentoring, and high involvement work practices) should be organized.

Additionally, a separate suitable rewards system might be designed for displaying supportive behaviors and looking for appropriate ways to reduce unavoidable exposure to others’ ostracizing behaviors. Performance evaluations must encompass anticipations of teamwork, knowledge sharing, and collaboration. Managers, leaders, and personnel must be answerable in case of failure to embrace the expectations of pleasant work atmosphere. Managers might not be able to prevent all exclusionary or hostile actions, but this study proposes that proactively producing the values that nurtures care, respect, and support can assist the personnel in handling stress. Providing the necessary means, channels, instruments, and tools to nurses for reporting incidences of covert mistreatment and an ongoing monitoring and evaluation are vital to avert and lessen the occurrence of ostracism that results in burnout, stress, and patient negligence in the nursing profession. Since nursing is dominated by female employees, this segment must be briefed about the effects of covert mistreatment and how it can put interpersonal relations at stake in work settings and on increasing stress levels with consequences for themselves as well as their patients.

In addition to these general suggestions to capture and reduce instances of WPO, this study is also particularly relevant to organizations where complete elimination of ostracism is not possible (De Clercq et al., 2018). There are situations where adverse behavior and mistreatment cannot be eliminated completely, like where organizational culture is categorized by overwork, high levels of intricacy, deep internal competition, or detached decision making (Pearson and Porath, 2005; Estes and Wang, 2008). This study showed that POS represents a positive job resource that makes employees feel that they are in a better position to deal with perceptions of WPO. Such employees are less prone to stress as they feel that they can count on their organization for assistance in their well-being. Organizations should continually strive to provide support in the form of fairness perceptions, organizational rewards, equity, work–family balance, and job conditions that can reduce unnecessary stress regarding the organizational functioning.

Additionally, organizational support might also be extended to employees in the form of facility of psychological counseling, if they come across any kind of workplace mistreatments. Anonymous support help lines might be useful for personnel to report their grievances and concerns without any fear (Rai and Agarwal, 2018). Moreover, coaching might bear fruitful results in training the employees to feel safe and supported while articulating their opinions and contributing toward organizational goals. This might further lead to team orientation and decrease in the incidences of WPO faced by the employees.

### Limitations and Future Directions

The limitations of the present research provide opportunities for further studies in the area. First, we concentrated on the moderating influence of a job resource, i.e., POS, on the relationship between WPO and stress; continued studies could add more moderating variables like self-efficacy (SE) and trait competitiveness.

Next, based on COR theory, the current study built on the premise that WPO would deplete resources; however, it did not explore the black box of WPO in more detail by studying other mediating pathways. According to Xia et al. (2019), WPO might induce stress and effect employee’s behaviors through multiple pathways, like hurting emotional as well as physical resources. Halbesleben et al. (2014) criticized that “it (i.e., only measuring the outcomes of resources loss or gain, such as stress) is not clear which resources are responsible for the change.” Moreover, environmental/job resources might only buffer the negative impact of ostracism on a particular resource (like stress). For example, Xia et al.’s, (2019) study has shown that spousal support can mitigate the association between WPO and emotional energy but not physical strength. Keeping this in mind, similar studies might be extended in the future by utilizing other mediating pathways between indirect relation of WPO and job-related outcomes including service sabotage. In addition to general POS, similar models might be replicated by including specific forms of POS (e.g., family supportive organizational perception) and general as well specific forms of SE (e.g., SE for work–family conflict management, occupational SE) as a personal resource.

Additionally, cross-country investigations might offer profound understanding into the comparative significance of unpleasant workplace environments for leveraging pertinent job resources for better work performance across diverse cultural settings. The study might be replicated in other provinces and industries from Pakistan and other countries to confirm the generalizability of the results.

It is also suggested to test other kinds of workplace maltreatments, like servant leadership, supervisor abuse, victimization, petty tyranny, workplace bullying, supervisor aggression, harassment, and negative mentoring experiences (Tepper, 2007; Hershcovis, 2011; Jahanzeb and Fatima, 2017). Interestingly, the authors were not able to locate many empirical studies associated to the antecedents of WPO. Future studies may use WPO as a mediating or outcome variable. This will permit organizations to examine the antecedents of WPO and consequently would be in a better position to recognize and decrease its incidence. Lastly, the negative effects of WPO on organizational consequences have been studied extensively; however, the literature is still deficient on its effects on employee’s family life and deserves further research attention.

## Conclusion

The stress that nurses come across during work interaction with colleagues can lead to harmful outcomes toward patient’s safety and can result in significant medical mistakes that might turn out to be detrimental for patients and hospital-related organizational consequences. Our research model showed that though WPO is related to stress, POS as moderator and positive job resource can lessen the strength of this association. The results have validated that POS represents an important work-related resource that makes personnel less prone to stress since they trust their organization as support of their well-being. Analysis endorsed the hypothesized relations and showed the moderating role of POS in the relation between WPO and stress. Our research has also contributed to the growing body of knowledge regarding POS (Rhoades and Eisenberger, 2002; Eisenberger and Stinglhamber, 2011). The findings demonstrate that by advancing supportive organizational perceptions, healthcare centers can assist personnel to feel cherished as organizational members and complete job tasks effectively even when relations at the workplace may not be ideal. Ensuring a supportive work culture would principally lie on the shoulders of leaders, managers, supervisors, and HR experts (Rhoades and Eisenberger, 2002), who may utilize POS to alleviate the harmful effects of WPO.

## Data Availability Statement

The datasets generated for this study will not be made publicly available due to the sensitive nature of the variables of study. The participants were ensured that full confidentiality would be maintained and data (including raw information) would not be shared at any platform.

## Ethics Statement

Ethical review and approval was not required for the study on human participants in accordance with the local legislation and institutional requirements. The patients/participants provided their written informed consent to participate in this study.

## Author Contributions

AS contributed in the design, write-up, data collection and analysis. MA supervised the whole study. HH aided in the write-up and data collection. MC helped in the data collection and analysis portion. All authors read and approved the final version of manuscript.

## Conflict of Interest

The authors declare that the research was conducted in the absence of any commercial or financial relationships that could be construed as a potential conflict of interest.
